# Infrared Thermography Reveals Sex-Specific Responses to Stress in Mice

**DOI:** 10.3389/fnbeh.2020.00079

**Published:** 2020-05-25

**Authors:** Jamshid Faraji, Gerlinde A. S. Metz

**Affiliations:** ^1^Canadian Centre for Behavioural Neuroscience, University of Lethbridge, Lethbridge, AB, Canada; ^2^Faculty of Nursing & Midwifery, Golestan University of Medical Sciences, Gorgan, Iran

**Keywords:** sex differences, infrared thermography, restraint stress, HPA axis, vertical activity deprivation, stress response, psychogenic fever, psychogenic hyperthermia

## Abstract

Psychogenic hyperthermia is a stress-related condition reported mostly in women. Neuroendocrine responses to stress in females differ from those in males, and these differences cannot be explained solely based on hypothalamic-pituitary-adrenal (HPA) axis activity. Here, we used infrared (IR) thermographic imaging to record changes in cutaneous temperature following two types of stressful experiences in female and male mice. Mice were exposed to either single-session restraint stress or vertical exploration (rearing) deprivation and were monitored for exploratory activity and IR surface thermal changes. Females displayed higher rearing activity than males during the dark phase of the light cycle. Both sexes showed similar plasma corticosterone (CORT) responses after a challenge with restraint and rearing deprivation. However, only females responded to rearing deprivation with increased cutaneous temperature in the head and back, and a reduced thermal response in the tail. Circulating CORT levels were not correlated with the thermal variations. These findings, for the first time, provide evidence for sex-specific cutaneous thermal responses to short-term stress in mice following transient vertical-activity deprivation that may mimic clinical psychogenic hyperthermia.

## Introduction

Intrinsic factors, as well as type, duration, and intensity of stress, determine the variation in physiological and behavioral stress responses. Sex is a key biological factor that influences vulnerability to psychological stress (Wang et al., 2007; Goldstein et al., 2010; Bale and Epperson, 2015). Thus, sex-dependent variation in stress response is a critical determinant of vulnerability to stress-related psychiatric disorders (Bangasser and Wiersielis, 2018). Nevertheless, preclinical studies mainly used males in the study of neurobiological and mental health correlates of stress vulnerability (Beery and Zucker, 2011). Moreover, studies using both sexes in rodents revealed robust sexual dimorphisms in psychoneurological responses to stressful challenges (Lin et al., 2009; Yamaura et al., 2013; Goodwill et al., 2019). For instance, in response to stressful experiences, hypothalamic signals activate the medullary raphe sympathetic premotor neurons to develop psychological stress-induced hyperthermia (PSH; Kataoka et al., 2014; Nakamura, 2015), a major psychosomatic symptom especially observed in young women (Petersdorf and Beeson, 1961; Weinstein, 1985; Oka, 2015).

Emotions experienced during transient or persistent stress, tension, anxiety, and fear may change body temperature in humans (Vinkers et al., 2013; Engert et al., 2014) and animals (Vianna and Carrive, 2005; Gjendal et al., 2018), thus proposing body temperature variations as a putative physiological marker of stress vulnerability and resilience (Lecorps et al., 2016). The neuroendocrine responses to stress by the hypothalamic-pituitary-adrenal (HPA) axis differs between females and males (McCarthy et al., 2012; Goel et al., 2014; Oyola and Handa, 2017), however, mere HPA axis activity does not fully explain the complex physiological phenotype of thermal responses to stress. Although mostly for males, preclinical findings portray a complex picture of stress-induced hyperthermia in fish (Rey et al., 2015), birds (Nord and Folkow, 2019), mice (Groenink et al., 1994; Olivier et al., 2003; Veening et al., 2004), and rats (Dallmann et al., 2006; McGivern et al., 2009). In humans, the influential impact of social status and higher cognitive processes further complicate the sex-dependent responses to stress (Handa and McGivern, 2009; Bale and Epperson, 2015). Due to local vasoconstriction and changes in cardiovascular activity, acute stress may reduce body temperature in the periphery without effects on the proximal core temperature (Vinkers et al., 2013). Facial temperature distribution shows a particularly sex-specific temporospatial pattern (Vinkers et al., 2013).

As a non-invasive imaging technique, infrared (IR) thermography (Tattersall, 2016) provides a robust assessment of surface thermal changes in living organisms. Although this technique has particular translational value for stress studies, it has not yet been extensively used in laboratory rodents. The present experiment was designed to examine sex differences in the cutaneous temperature during two types of short-term stress in mice along with biological and behavioral markers of HPA axis activation. The stress paradigms were based on the restriction of multi-dimensional restraint stress and restriction of vertical exploration only (rearing deprivation). Recent findings showed that rearing deprivation challenges HPA axis activity and emotional status (Faraji et al., 2016). Here, the results showed elevated plasma corticosterone (CORT) levels in response to both types of stress in both females and males. However, females experienced greater thermal changes than males only when deprived of their rearing behavior. The findings, for the first time, provide evidence for sex-specific cutaneous thermal response in mice following transient rearing deprivation that may mimic clinical psychogenic hyperthermia.

## Materials and Methods

### Animals

Adult male and female mice (C57BL/6NJ; B6), 3–4 months old, were used in this study. The animals were housed in pairs under a 12:12 h light/dark cycle with the light starting at 07:30 h. Animals were provided with water and food *ad libitum*. The room temperature was set at 22°C, and experimental procedures were conducted during the light phase of the cycle at the same time of day. All animals were handled for approximately 5 min daily for three consecutive days before any experimental manipulations. All procedures were approved by the University of Lethbridge Animal Care Committee in compliance with the standards set out by the Canadian Council for Animal Care (CCAC).

### Experimental Design

**Figure 1:**
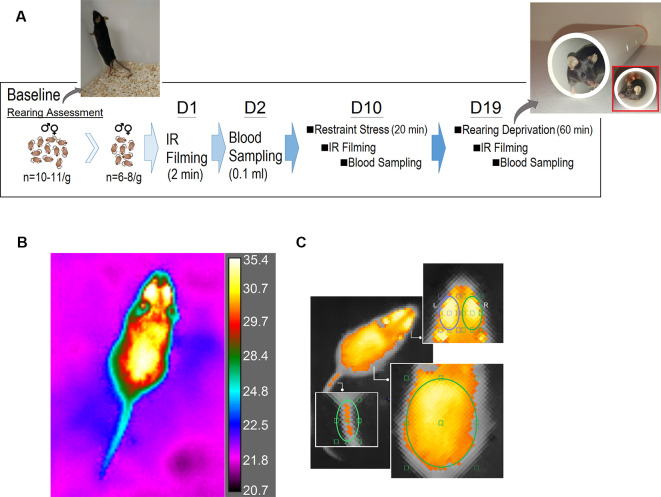
Experimental design. **(A)** Time course illustrating the experimental manipulations. A customized white PVC pipe was used to restrict vertical exploration (rearing). The tunnel allowed animals to freely move and turn around in the tunnel but prevented rearing on their hind limbs. **(B)** A representative bird’s eye view plot of a freely moving mouse using the FLIR infrared (IR) thermographic camera. The camera was placed 80 cm above the animal, and was able to follow changes in the animal’s surface temperature (*emissivity*: 0.98, *thermal resolution*: 320 × 240 pixels per image, *thermal sensitivity*: <30 mK at 30°C, *image frequency*: 60 HZ). **(C)** Thermographic imaging showing four regions of interest [head (left and right), back and base of tail] in assessments of surface temperature before and after restraint and rearing deprivation stress.

Figure 1A illustrates the time course of experimental manipulations. Pre-stress (baseline) number of rears was calculated in two groups of male and female mice (*N* = 11/group) for 5 min each during light (day) and dark (night) phase. Also, pre-stress assessment (day 1) of thermal changes was performed before stress using a square transparent Plexiglas box (25 × 25 × 30 cm). Mice (males, *N* = 8; females, *N* = 6) were individually placed in the box for 2 min, and their free movement was recorded with an IR camera. On day 2, animals underwent blood sampling for CORT measurements as the main physiological indicator of the HPA axis activation. Animals were then allowed to recover for 7 days.

On day 10, individual animals were subjected to a single session of restraint stress for 20 min immediately followed by IR thermal recording and blood sampling 3–4 min later. Animals were then given a second seven-day recovery period. On day 19 animals were given a single session of rearing deprivation for 60 min again immediately followed by IR thermal recording and blood sampling. The same procedures for IR recording and blood sampling were used for both post-stress sessions. All animals received equal amounts of handling daily throughout the experiments, and the data collection, thermal, and CORT assays were performed by two experimenters blind to the group identities.

### Assessment of Vertical Exploratory Activity

Two groups of randomly-selected male and female mice (*N* = 11/group) were monitored for rearing activity before any experimental manipulations. The number of rears during free exploration was recorded after placing animals into the middle of a square, black Plexiglas open-field arena (70 × 70 × 35 cm). The number of rears was counted for 10 min segregated into two 5-min for the day and nighttime (8:30–9:30 am and 9:00–10:30 pm), both under dim illumination. Vertical exploration (rearing) was scored when mice reared on their hind limbs regardless of whether rears occurred on or off the walls (supported and unsupported rearing; Sturman et al., 2018). Animals typically appear stationary with slow or absent whisker movement in rears (Faraji et al., 2013, 2016). The movements of the animals were recorded and analyzed by an HVS tracking system (HVS Image, UK), and rears and stops were analyzed and illustrated by a motion graph software (SINA Motiongraph, V.II, Iran).

### Infrared (IR) Imaging

A FLIR IR thermographic camera (FLIR T450sc, Sweden; fixed emissivity = 0.98 specified for skin in the manufacturer’s emissivity table) mounted on top of a transparent Plexiglas box (20 × 20 cm) recorded temperature in freely moving mice for 2 min per test session. IR imaging occurred in a windowless room with a steady temperature set at 22°C and relative humidity of ~50%. Animals were protected from direct ventilation. The post-stress thermal imaging was performed immediately (~1 min) after removing animals from the restraint tube or rearing a deprivation tunnel.

Briefly, animals were placed individually in the center of the Plexiglas box with the dorsal side up, and the IR thermographic recording was performed above the box without a lid because IR radiations are blocked by Plexiglas or stainless steel. The camera was placed 80 cm above the animal and was able to follow changes in the animal’s surface temperature and its immediate surrounding with a thermal resolution of 320 × 240 pixels per image, thermal sensitivity of <30 mK at 30°C, and 60 Hz acquisition rate (Figure 1B).

IR thermal profiles were then saved and analyzed using the FLIR image processing software (FLIR ResearchIR Max software 4.40.6.24). For the thermal analysis, three regions of interest (ROIs) were chosen. (1) *Head* (including eyes), covering a major portion of the frontal and parietal surfaces. An approximate measure of the sagittal suture allowed the elliptic ROI to split the top of the head up into the left and right sides. Also, a small segment of interparietal bone was included in either right or left ROIs. To control the effect of the position of each animal on the emitted thermal irradiations, the best postural condition for the head was chosen when mice were moving with their head oriented straight ahead without deviation to sides. (2) *Back*, an oval-shape ROI included lower thoracic and upper and lower lumbar levels extended to the abdominal parts at equidistance from approximately 1.5 cm off the midline. (3) *Tail* (base), where the base of the tail joins the rump. In total, four ROIs [head (left and right), back and tail] were considered for analysis of changes in surface temperature (Figure 1C).

For sampling, six frames representing six time-bins (20–30, 35–45, 50–60, 65–75, 80–90, 95–105 s) adjusted to the corresponding ROIs from the head and back, and two frames (30–60, 90–120 s) from the tail base were chosen for each animal. ROI sizes were identical for all frames and mice. The best-fit area to the ROIs in each frame/time bin was determined based on the animal’s dorsal posture when approximately all relevant regions were bounded by the radius of the ellipses and/or when the animal was found in a prone position with all four limbs on the ground.

### Stress Procedures

#### Multi-dimensional Restraint

A single-session of classic restraint stress was performed for 20 min (Zucchi et al., 2014; Ambeskovic et al., 2017; Jafari et al., 2017). Male and female mice (*N* = 6–8/group) were individually placed into custom-made transparent Plexiglas tubes (3 cm inner diameter) of adjustable length. The tubes maintained the mouse in a standing position without compression of the body. Restraint occurred in a designated quiet, semi-dark room maintained at 22°C.

#### Rearing Deprivation (Vertical Restraint)

A single session of vertical activity deprivation was performed for 60 min. Each animal was placed in a white PVC pipe (5 cm inner diameter; 40 cm length) mimicking a natural burrow habitat of a mouse. The tunnel allowed animals to freely move and turn around but prevented rearing on their hind limbs. Windows inserted in the wall of the apparatus supported air ventilation [(Faraji et al., 2016) with modification]. The stress occurred in a designated quiet, semi-dark room maintained at 22°C.

The duration of the restraint stress and rearing deprivation protocols (Faraji et al., 2016; Jafari et al., 2017) in the present experiment were determined based on previous pilot observations in mice indicating that the stress response after 20-min restraint stress (RS) is similar to 60-min rearing deprivation (RD).

### Blood Sample Collection and Corticosterone Measurements

Blood samples were taken a week before and 3–4 min after stress procedures to assess circulating CORT levels. Mice were restrained by grasping the loose skin over the shoulder and behind the ears where the skin was taut over the mandible. A small puncture was made on the sub-mandibular vein using a lancet. Approximately 0.1 ml of blood was collected with a micro-tube. All samples were collected in the morning hours between 9:00 and 11:00 AM. Animals were returned to their home cage and allowed for recovery for 1 week. Plasma was obtained by centrifugation at 5,000 rpm for 10 min. The plasma samples were stored at −80°C and analyzed for CORT concentration using commercial ELISA kits (Abcam, Toronto, ON, Canada).

### Statistical Analysis

All data were analyzed with an SPSS 16.0 package (SPSS Inc., USA). Effects of main factors [Group—two levels; Sex—two levels; Time—two levels (night and day); Time bin—six levels; Session—three levels; Regions—three levels] were analyzed as independent variables for the number of rears, the surface temperature in different ROIs and plasma CORT as dependent variables by *repeated measure*, *one*- and *two-way* ANOVA. Statistical differences were determined with Tukey’s HSD multiple comparisons *post hoc* test (adjusted *p*-values are shown where appropriate). To test for correlations between the level of circulating CORT and surface temperature Pearson product-moment correlation coefficient was applied. Separate means comparison between groups was also performed using dependent and independent samples *t*-tests for within-subject comparison where applicable. The Familywise error was considered before the multiple *post hoc* analyses if necessary. To evaluate the magnitudes of the effects of experimental manipulation (here, restraint, and rearing deprivation) on the body surface temperature, effect sizes (*η*^2^ for ANOVA) were calculated. Values of *η*^2^ = 0.14, 0.06, and 0.01 were considered for large, medium, and small effects, respectively. A *p*-value of less than 0.05 was considered statistically significant. Data are presented as the mean ± SEM.

## Results

### Number of Rears

**Figure 2:**
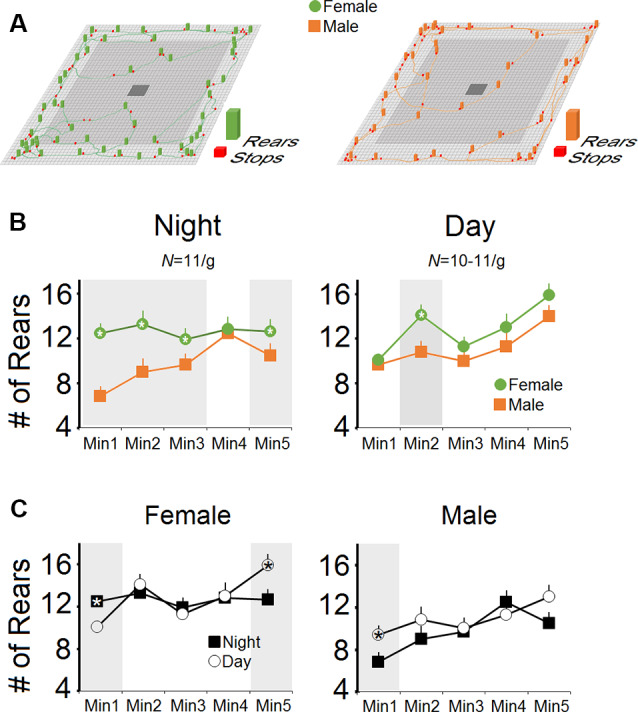
Rearing activity in female and male mice during the night- and daytime hours. **(A)** Representative exploratory locomotor trajectories of a female and a male mouse in the nighttime along with rears and stops during free exploration in an open field arena. Females displayed more rears at nighttime (dark phase) exploration than males. **(B)** The number of rears during minutes 1–3 and minute 5 of the test session during nighttime in females was significantly higher than males. In contrast, female mice performed more rears than males only in the second minute of exploration during daytime (light phase). **(C)** When the number of rears during the night- and daytime were compared, both sexes showed similar patterns of rearing activity. Gray box demarks the statistically significant differences.

Figures 2A–C show the number of pre-stress rears as assessed in male and female mice during the day- and night-time. There was a main effect of Group (*F*_(1,20)_ = 9.77, *p* = 0.005, *η*^2^ = 0.32; *repeated measure* ANOVA) and Group × Time interaction (*p* = 0.041). Time (*p* = 0.13) and Time Bin (*p* = 0.072), as well as Group × Time Bin (*p* = 0.055) in terms of the number of rears at night, indicated that over a 5-min period of assessment, females made more rears than males (63.09 ± 5.33 vs. 48.36 ± 5.33). Furthermore, females displayed a higher rate of rears than males in each time bin (all *p* < 0.05, *one-way* ANOVA; Figure 2B), excluding the fourth minute.

However, the main effect of Group for the rears in the daytime was not significant (*p* = 0.059), although the significant effect of Time bin indicated that females and males showed the same progressive rate of rears as the exploration proceeded in the daytime (64.36 ± 4.76 vs. 55.72 ± 4.76). No Group × Time Bin interaction was observed (*p* = 0.55). Also, there were no significant differences between the number of rears in the day- and nighttime in each group (both *p* > 0.05; *repeated measure* ANOVA) whereas *one-way* ANOVA revealed significant differences between rears only in the first and last minutes of exploration in females and only the first minute of exploration in males (all *p* < 0.05; Figure 2C). In summary, no group differences were observed between day and night, even though females displayed more rears than males at night during the dark phase.

### Circulating Plasma CORT Levels

**Figure 3:**
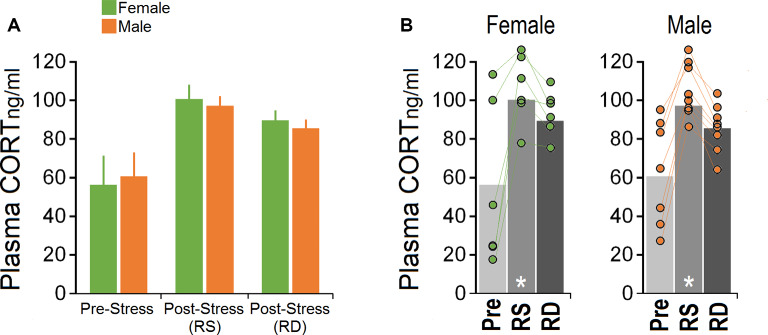
Corticosterone (CORT) levels in response to acute restraint and rearing deprivation in female and male mice. **(A,B)** Both sexes exhibited significantly greater hypothalamic-pituitary-adrenal (HPA) axis reactivity to RS compared to RD. Females and males responded equally to rearing deprivation. Circles represent individual mice in each group and experimental session. There was no significant difference between pre-stress and post-RD CORT levels. White asterisk: significance relative to pre-stress session, *p* ≤ 0.05; *one-way* ANOVA, *N* = 6–8/group. Pre, pre-stress; RS, restraint stress; RD, rearing deprivation.

One male mouse was excluded from the CORT analysis in the pre-stress session due to technical issues. Figures 3A,B illustrate CORT levels in females and males across sessions. No significant effect of Group (females vs. males; *p* = 0.764, *repeated measure* ANOVA) but a significant effect of Session (pre-stress vs. post-stress restraint vs. post-stress rearing deprivation; *F*_(2,24)_ = 13.75, *p* = 0.000, *η*^2^ = 0.534; *repeated measure* ANOVA) was observed. The Group × Session interaction was not significant. Within-group comparison for the main effect of Session by *one-way* ANOVA showed a significant effect of Session (*F*_(2,17)_ = 4.29, *p* = 0.033) only in females where they displayed higher levels of CORT in response to both restraints (100.98 ± 6.70 ng/ml) and rearing deprivation (86.68 ± 4.42 ng/ml) compared to pre-stress (54.53 ± 18.14 ng/ml; all *p* < 0.05; *post hoc* Tukey). Furthermore, a similar CORT response to restraint and rearing deprivation was observed in males (*F*_(2,23)_ = 10.62, *p* = 0.001; pre-stress: 58.74 ± 7.75 ng/ml, post-stress restraint: 94.28 ± 10.99 ng/ml, post-stress rearing deprivation: 82.48 ± 4.15 ng/ml). *Post hoc* Tukey only showed a significant difference between pre-stress CORT and post-stress restraint (*p* < 0.001). Overall, changes in circulating CORT indicated that the HPA axis responded similarly to stress in female and male mice. Also, females showed more exaggerated HPA axis responses than males although this was not significant.

### IR Surface Temperature

#### Pre-stress Session

**Figure 4:**
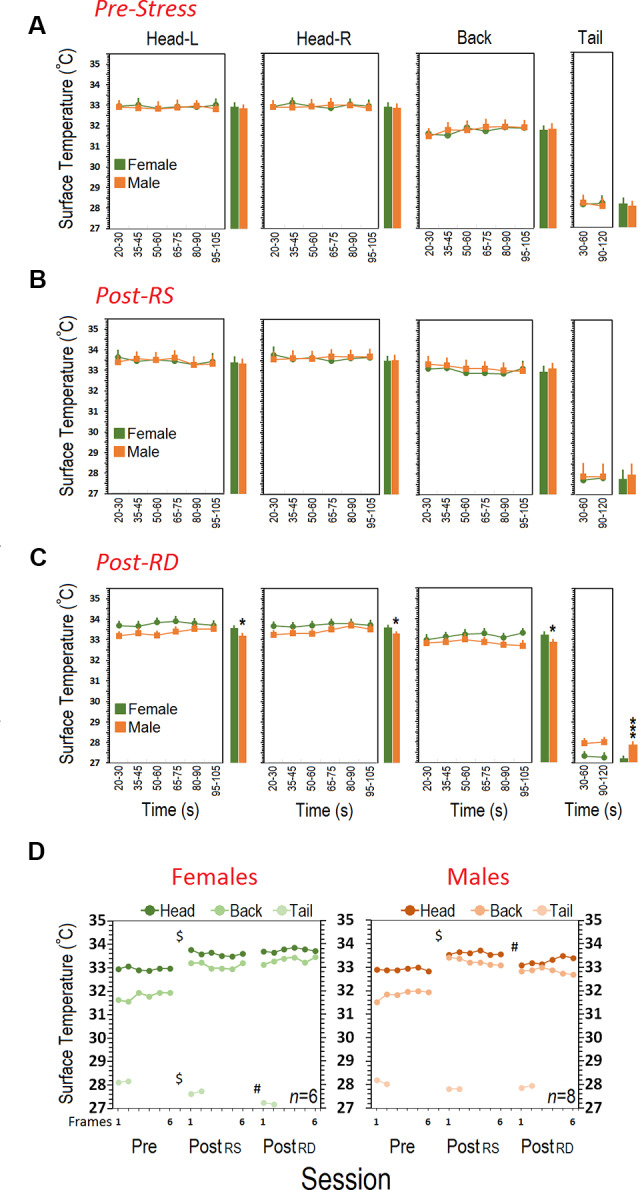
Comparison of cutaneous thermal changes in different regions of interest. **(A)** Pre-stress thermal responses in females and males during a 2-min session of free exploration. **(B)** Females and males responded to restraint stress in the post-stress RS phase with an enhanced surface temperature in the head and back. Restraint stress, however, reduced cutaneous thermal responses in the tail of females. **(C)** Patterns of thermal responses during rearing deprivation in females post-stress RD were significantly different than those of male mice. Surface temperature in the head and back of females significantly increased whereas it decreased in the tail when compared with males. The individual bar graphs next to each panel depict the average surface temperature for each group. **(D)** While thermal responses in females to restraint and rearing deprivation did not differ, rearing deprivation in males reduced thermal responses in the head and back close to pre-stress levels. ^$^Pre-stress vs. Post-RS and Post-RD; ^#^Post-RD vs. Post-RS and Pre-stress; *p ≤ 0.05, ***p ≤ 0.000; *one-way* and *repeated-measure* ANOVA, *N* = 6–8/group.

Figure 4A shows the mean surface temperature of the head (left and right), back, and tail for each group in the pre-stress session before any experimental manipulation. Both groups (*N* = 6–8) displayed a similar pattern in cutaneous temperatures across all six-time bins. *Repeated-measure* ANOVA for head did not show significant effects of Group (female vs. male), Side (left vs. right), and Time Bin (all *p* > 0.05). However, a significant effect of Region (*F*_(2,15)_ = 9.02, *p* = 0.03, *repeated-measure* ANOVA) indicated that the heat change pattern in the ROIs appeared to follow an order of head > back > tail where the head in both groups emitted significantly more heat (female, 32.96 ± 0.07°C vs, male, 32.90 ± 0.06°C) than back (female, 31.80 ± 0.16°C; male, 31.85 ± 0.13°C) and tail (female, 28.14 ± 0.17°C; male, 28.10 ± 0.15°C; all *p* < 0.05, *post hoc* Tukey).

#### Post-stress Restraint

Thermal changes influenced by a 20-min single session of restraint stress are illustrated in Figure 4B. Restraint stress increased the surface temperature of the head and back in both groups (*N* = 6–8). *Repeated-measure* ANOVA did not reveal significant effects of Group and Side (all *p* > 0.05) indicating that females and males responded similarly in the left and right sides of the head (female, 33.60 ± 0.07°C vs. male, 33.60 ± 0.06°C) and back (female, 33.09 ± 0.16°C vs. male, 33.23 ± 0.13°C). Furthermore, the average tail temperature in females for both time bins was slightly lower than males (female, 27.68 ± 0.17°C vs. male, 27.81 ± 0.15°C) even though this was not significant (*p* > 0.05).

#### Post-stress Rearing Deprivation

Results showing the thermal changes affected by a 60-min rearing deprivation in male (*N* = 8) and female (*N* = 6) mice are shown in Figure 4C. The heat emitted from all ROIs presented a robust effect of Sex (Group). Similarly to the HPA-axis response, the cutaneous thermal changes in females after rearing deprivation revealed significantly higher temperatures than males in the head (female, 33.75 ± 0.07°C vs. male, 33.40 ± 0.06°C; *F*_(1,12)_ = 7.09, *p* = 0.02; *η*^2^ = 0.37, *repeated-measure* ANOVA) and back (female, 33.33 ± 0.12°C vs. male, 32.97 ± 0.10°C; *F*_(1,12)_ = 4.82, *p* = 0.04; *η*^2^ = 0.28, *repeated-measure* ANOVA). No effects of Side (left vs. right), Side × Sex, and Side × Time Bin were found.

**Figure 5:**
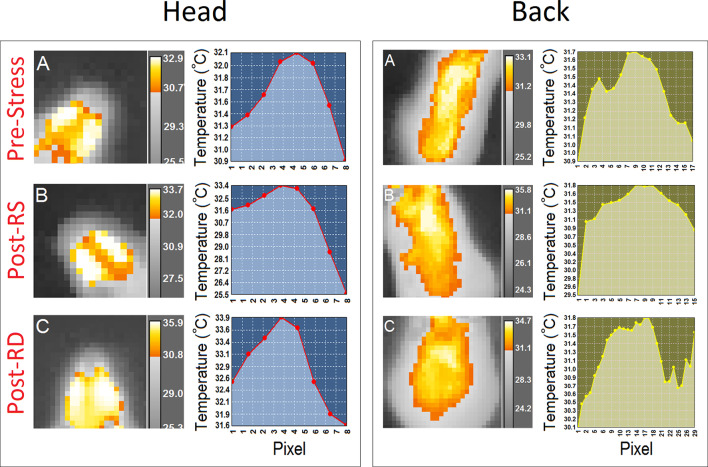
Thermal responses to acute restraint and rearing deprivation stress in a female mouse. Panels **(A–C**; left panels**)** provide samples of thermographic images of the head and back in a female mouse during the second time bin (35–45 s) for **(A)** pre-stress, **(B)** post-stress restraint, and **(C)** post-stress rearing deprivation. Thermographic images were taken with an infrared thermographic camera positioned above the animal during free open field exploration. The different color-coding for temperature was extracted from the FLIR image processing software. Also, thermographic variations in all three experimental conditions are depicted by the profile plots in the right panels.

The profile of thermal changes in the tail of females was significantly different from males (female, 27.21 ± 0.08°C vs. male, 27.91 ± 0.07°C; *F*_(1,12)_ = 39.28, *p* = 0.000; *η*^2^ = 0.76, *repeated-measure* ANOVA) indicating that females displayed reduced thermal responses than males. There was no effect of Time Bin nor a Time Bin × Sex interaction. Furthermore a significant effect of Session (pre-stress vs. post-stress RS vs. post-stress RD) followed by *post hoc* Tukey analysis indicated that females displayed an exacerbated thermal response to the rearing deprivation in all ROIs relative to males (all *p* < 0.05) when compared with pre-stress and post-stress restraint sessions (all *p* < 0.05; Figure 4D). The effect of Region (head vs. back vs. tail) was significant for all sessions (all *p* < 0.05, *repeated-measure* ANOVA). Figure 5 compares thermal changes in the head and back of one female animal across experimental sessions.

In summary, even though both sexes experienced similar degrees of cutaneous thermal change in all three ROIs in response to restraint stress, the additional comparative investigation revealed that females are more vulnerable to the rearing-deprivation stress than males as shown by increased surface temperature in the head and back (Supplementary Movies S1, S2), and a decreased thermal response in the tail.

### Correlational Analysis

Because there were no differences in the thermal response between the left and right sides of the head, and no effect of Time Bin, the average of cutaneous temperatures for the left and right head were used for correlational analysis. Correlational analysis revealed no significant relationship between plasma CORT and changes in surface temperature in the corresponding ROIs in either of the groups [Pre-Stress (males): Head *p* = 0.661, Back *p* = 0.864, Tail *p* = 0.023; (females): Head *p* = 0.189, Back *p* = 0.156, Tail *p* = 0.609; Post-RS (males): Head *p* = 0.595, Back *p* = 0.787, Tail *p* = 0.342; (females): Head *p* = 0.542, Back *p* = 0.763, Tail *p* = 0.53; Post-RD (males): Head *p* = 0.999, Back *p* = 0.637, Tail *p* = 0.985; (females): Head *p* = 0.687, Back *p* = 0.230, Tail *p* = 0.380].

## Discussion

The present study provides the first description of sex-dependent thermal responses to acute experimental stress in a mouse model. Using two single-session stress paradigms, we examined sex differences in surface temperature variations in response to multi-dimensional restraint or rearing deprivation. Both sexes showed similar levels of HPA-axis activity in response to restraint stress and rearing deprivation. However, females experienced greater surface thermal changes than males after being temporarily deprived of their rearing behavior. Thus, results may contribute to uncovering the multifaceted nature of stress responses in mice along with sex-dependent thermoregulatory function in response to acute stress, arguably addressing a preclinical model for PSH.

Rearing, during which rodents intermittently discontinue their horizontal activity and rear up by lifting one or both forelimbs from the ground, usually involves emotional and processing components during spontaneous motor behavior (Gironi Carnevale et al., 1990; Lever et al., 2006). Two types of rearing behaviors, supported vs. unsupported, can be distinguished, each representing characteristic ethological features of exploration (Sturman et al., 2018). Structural correlates of rearing behavior also indicate that vertical exploration is closely linked, for instance, to hippocampal morphology (Roullet and Lassalle, 1990; Hausheer-Zarmakupi et al., 1996; Thiel et al., 1999; Boulle et al., 2014; Faraji et al., 2016; Barth et al., 2018). These functional and structural findings predominantly emphasize the dynamic role of rearing in normal motor behaviors.

Following previous reports (Podhorna and Brown, 2002; Easton et al., 2003; Augustsson et al., 2005), females in the present experiment tended to rear more than males, particularly during nighttime. Greater rearing activity in females may not strictly reflect emotional salience but may also be attributable to higher levels of anxiety and higher risk assessment (Lever et al., 2006). Thus, rearing movements lack predictive value for emotional status. The deprived male rats also emitted a greater number of distress calls (22-kHz ultrasonic vocalizations) and spent significantly more time vocalizing than their non-deprived counterparts (Faraji et al., 2016). If this is the case, it seems justified to hypothesize that rearing deprivation is associated with emotional disturbances in mice. Accordingly, in the present experiment, we sought sex differences in response to single-session rearing deprivation in mice using plasma CORT levels and IR thermography.

HPA axis hyperactivity is a characteristic aspect of responses to either transient or repeated stressful experiences in rodents, which is typically accompanied by increased circulating CORT concentration (Markham et al., 2006; Gourley et al., 2013; Browne et al., 2014; Faraji et al., 2014, 2017). Higher hormonal output in the female stress response particularly during short-term stress (Goel and Bale, 2008; Sterrenburg et al., 2012) not only indicates that their HPA axis function initiates more rapidly than that of males (Iwasaki-Sekino et al., 2009) and remains elevated for longer (Jadavji and Metz, 2008), but also shows that females are endowed with a natural hormonal capacity to regulate stress responses that differs from males (Rhodes et al., 2001; Goldstein et al., 2010). Nevertheless, the elevated CORT levels indicated that the temporary lack of horizontal activity equally activated the HPA axis in both sexes, however, the slightly higher response in females relative to males is in parallel with previous reports (Iwasaki-Sekino et al., 2009).

Rearing deprivation may also differentially regulate HPA axis activity depending on stress duration. While presently the mice failed to display a significant impact of acute rearing deprivation on CORT levels, our previous findings in rats showed elevated CORT level only in response to chronic but not acute rearing deprivation (Faraji et al., 2016). Accordingly, stressor duration and intensity determine the behavioral response (see Sandi and Pinelo-Nava, 2007 for review), and so the effects of single-session acute rearing deprivation differ from the repeated prolonged deprivation used in our previous work with rats. Further discrepancies to the previous literature might derive from the potential interference of the estrus cycle with the females’ HPA-axis responsivity, which was not systematically investigated in the present experiment. The present strategy for randomization of sample selection and group assignment might diminish confounding effects of the estrous cycle, however, conclusions about HPA-axis activity during both stress paradigms in the absence of estrous cycle monitoring should be drawn with caution and warrant further consideration. Although supported by experimental evidence (Herman et al., 2016), the use of different stress durations (60-min RS vs. 20-min RD) challenges the comparability of HPA axis activation and CORT responses and may confound conclusions about the two stressors. This caveat should be carefully addressed in future investigations.

In contrast to CORT levels, the cutaneous temperature was more responsive to stress, particularly in females. We also compared all thermal outcomes with CORT concentration in search of sex differences in thermal responses to stress. Like the HPA-axis response, both groups displayed identical patterns of thermal variations during the pre-stress and post-stress restraint sessions. There was a similar increase in head temperature in both sexes after exposure to restraint when compared to the pre-stress phase, although the thermal response in the back and tail was slightly higher in males. When exposed to rearing deprivation, however, females showed higher surface thermal variations in most ROIs (except for the tail) than did males. Interestingly, this sex-dependent thermal response to the rearing deprivation was not correlated with circulating CORT concentration indicating a distinct neuroregulatory system in thermal response to emotionally threatening stimuli.

The peripheral autonomic nervous system, which underlies heart rate and breathing, tissue metabolism, respiration, and surface blood perfusion, also determines biological heat emission (Ioannou et al., 2014) during short-term aversive experiences. Hence, IR thermal imaging of cutaneous temperature (Vianna and Carrive, 2005; Lecorps et al., 2016; Tattersall, 2016; Fiebig et al., 2018; Gjendal et al., 2018) may provide a sensitive measure of an organism’s agitation and emotional response, regardless of their HPA axis response. Concerning the noticeable decrease in tail temperature along with a significant increase in the head (including eyes) and back temperatures, it appears that mainly females display the classic symptoms of thermal activity in response to stressful events (Vianna and Carrive, 2005; Lecorps et al., 2016). The contradictory aspects of thermal maps in females (i.e., the marked drop in tail temperature vs. the noticeable increase in head and back temperature) may reflect a sex-specific cutaneous vascular change of local shifts in vasoconstriction and dilation following rearing deprivation.

The emotional display is associated with the complementary patterns of vascular responses of vasoconstriction and vasodilation. Previously, it was suggested that the tail is the main part of the body for dissipating internal heat accumulated during an aversive emotional experience (Vianna and Carrive, 2005). The present findings show that female thermal maps are rather generalized across all body parts as opposed to males, which were not affected by rearing deprivation. Whether the enhanced temperature in these regions represents a low-magnitude sympathetic vasoconstrictor signal to skin arteries (Blessing, 2003; thus facilitating blood flow in the skin) or greater central processing (inducing a passive thermal change *via* blood circulation; Lim et al., 2008; Charkoudian and Stachenfeld, 2014) is beyond the scope of the present study. However, they reveal the sex-specific nature of thermoregulatory activity in mice in response to rearing deprivation. From an alternative viewpoint, sex-dependent thermal differences in response to rearing deprivation may also be attributable to the failure of adaptive physiological responses to acute stress in males. Considering the sex-biased significance of the vertical activity, however, sex differences in cutaneous thermal responses may rather reflect females’ vulnerability to the transient vertical-activity deprivation.

It may also be speculated that vertical activity in rodents contributes to core and skin temperature regulation particularly in females, as suggested by the impact of postural changes on surface temperature (Tikuisis and Ducharme, 1996; Van Someren, 2006). It is widely accepted that hormonal, such as estrogenic, influences are also involved in regulating core and skin temperature (Charkoudian, 2001; Charkoudian and Stachenfeld, 2014; Charkoudian et al., 2017). Accordingly, mainly females thermally responded to acute distress by regional cutaneous hyperthermia in the head and back along with hypothermia at the base of the tail. A similar contrasting pattern of topographical thermal changes was also reported for humans (Vinkers et al., 2013). A confound in these measures may be also caused by body weight and size differences. In rodents, it is difficult to minimize the effects of body size when investigating sexual dimorphisms in cutaneous thermal responses, especially considering the digressing trajectories with increasing age.

PSH is a major psychosomatic symptom which is also called functional hyperthermia (Oka, 2015) or psychogenic hyperthermia (Olivier, 2015) that occurs most frequently in young women (Weinstein, 1985; Oka, 2015). While hormonal impacts on thermal regulation in females are unequivocal, the underlying neurohormonal mechanism in stress-induced hyperthermia or hypothermia remains less clear. Here, we demonstrate a sex-specific topographical thermal response to acute stress in IR thermal imaging in mice. The contrasting profile of HPA axis activation and IR thermal variations in response to acute stress not only presents a dynamic indicator of sex-dependent patterns in rearing behavior but also suggests that CORT-independent mechanisms account for stress-induced hyper- and hypothermic responses. Thus, although they contradict the previous report (Groenink et al., 1994), the findings support the earlier notion that stress-induced emotionality in rodents is not merely based upon HPA axis hyperactivity and/or elevated plasma CORT (Faraji et al., 2011), as it may also occur through non-adrenocorticotropin-mediated mechanisms (De Souza and Van Loon, 1982; Duric and McCarson, 2005). The present observations emphasize the importance of using an array of multiple assessments to appreciate the complex physiological impact of stress on the phenotype. Thus, IR thermal imaging provides a feasible and non-invasive real-time measurement of psychophysiological functions following stress.

## Data Availability Statement

The datasets generated for this study are available on request to the corresponding author.

## Ethics Statement

The animal study and procedures were reviewed and approved by the University of Lethbridge Animal Care Committee in compliance with the standards set out by the Canadian Council for Animal Care (CCAC).

## Author Contributions

JF and GM designed the study. JF performed the experiments and analyzed the data. JF and GM wrote the article and approved the final version.

## Conflict of Interest

The authors declare that the research was conducted in the absence of any commercial or financial relationships that could be construed as a potential conflict of interest.

## Supplementary Material

VIDEO S1 Cutaneous thermal changes in different regions of interest in a representative stressed female mouse after rearing deprivation. Females were more vulnerable to the rearing-deprivation stress than males as shown by 1.47°C average increased surface temperature in the head and back.

VIDEO S2 Cutaneous thermal response to rearing-deprivation stress in a representative male mouse. Unlike females, males showed a similar thermal response to restraint stress and rearing deprivation.
